# Religio Medici, a Letter to a Friend, Christian Morals, Urn Burial, and Other Papers

**Published:** 1862-02

**Authors:** 


**Religio Medici, A Letter to a Friend, Christian Morals, Urn Burial and othe Papers. By Sir Thomas Browne, Kt., M. D., Boston: Ticknor & Fields. 1862.**

The house of Ticknor & Co. has done more to give us a good literature, by the sterling character of its books, its generous dealing with authors at home and abroad, and the artistic and mechanical perfection of its work, than any other house in our country. Their blue and gold books, started years ago with *Tennyson*, was a stroke of genius as rare as the power to write a poem; they could no more help being popular than the June roses can. Hosts of imitators bear testimony to their perfect beauty and fitness; and there is still only one house that can give us a blue and gold book.

This book is still better than any other yet printed, for our daily company, and it is just what we wanted. The old folios are buried in great libraries; the English octavos are very dear; Bohn's edition is of moderate price, but Sir THOMAS, like many a good man beside, wrote some as he was moved by the Holy Ghost, and some as he was moved by the publishers, and the present editor has carefully and reverently gathered one from the other. We suppose this octavo, in brown and crimson, 432 pages, printed on a generous paper, with a sweet portrait, quaint head and tail-pieces, exquisite initial letters, and running notes in the margin, is, to all other editions, what wheat clean sifted is to wheat in the stack. For some insight into the surpassing goodness of the book itself, the mingled wisdom, piety and learning, the curious blending of medical and religious suggestion, written in honest, terse old English, we must send the reader to the book. For the younger and rising men in the profession, especially, we can suggest no book likely to be at once so wholesome and so welcome, as this of the good physician; one of whose *Resolves*, found in his common-place book, we here transcribe:—

"To pray daily, and particularly for sick patients, and in general for others, wheresoever, howsoever, and under whose care soever; and at the entrance into the house of the sick, to say, The peace and mercy of God be in this place."

